# Magnetic Resonance Imaging Artifacts and Cochlear Implant Positioning at 1.5 T In Vivo

**DOI:** 10.1155/2018/9163285

**Published:** 2018-11-08

**Authors:** Dirk Schröder, Gloria Grupe, Grit Rademacher, Sven Mutze, Arneborg Ernst, Rainer Seidl, Philipp Mittmann

**Affiliations:** Department of Otolaryngology, Head and Neck Surgery, Department of Radiology, Unfallkrankenhaus Berlin, Germany

## Abstract

**Objective:**

Cerebral magnetic resonance imaging with the magnet of the cochlear implant receiver/stimulator in place causes artifacts and hinders evaluation of intracerebral structures. The aim of this study was to evaluate the internal auditory canal and the labyrinth in a 1.5T MRI with the magnet in place.

**Study Design:**

Observational study.

**Setting:**

Tertiary referral center.

**Subjects and Methods:**

The receiver/stimulator unit was placed and fixed onto the head of three volunteers at three different angles to the nasion–outer ear canal (90°–160°) and at three different distances from the outer ear canal (5–9 cm). T1 and T2 weighted sequences were conducted for each position.

**Results:**

Excellent visibility of the internal auditory canal and the labyrinth was seen in the T2 weighted sequences with 9 cm between the magnet and the outer ear canal at every nasion–outer ear canal angle. T1 sequences showed poorer visibility of the internal auditory canal and the labyrinth.

**Conclusion:**

Aftercare and visibility of intracerebral structures after cochlear implantation is becoming more important as cochlear implant indications are widened worldwide. With a distance of at least 9 cm from the outer ear canal the artifact induced by the magnet allows evaluation of the labyrinth and the internal auditory canal.

## 1. Introduction

Magnetic resonance imaging (MRI) has become a standard diagnostic procedure with different indications over all medical specialties and is part of the preoperative test battery for candidates for cochlear implant surgery. With over 350000 implantees worldwide, the probability of a MRI scan in an implanted patient for medical reasons is quite high [1]. The current rate of MRIs performed in cochlear implantees in Western countries is about 10%.

The indication for cochlear implantation has evolved in recent decades from single-sided implantation in bilateral patients, to bilateral implantation and to patients with residual hearing and asymmetric hearing loss. This widening of the indication range has increased the probability of postoperative scanning for different medical reasons.

With increasing numbers and availability of MRI scans, patients with vestibular or intracochlear schwannomas can gain from a cochlear implant. Such patients undergo either primary surgery and subsequently implantation as a single-step [2, 3] or a two-step procedure within a controlled time-frame in order to decrease the probability of tumor recurrence [4].

MRI artifacts at 3T that are induced by the cochlear implant have been reported to make it extremely difficult to realistically assess audiovestibular structures [5] with a CI magnet in place. Nevertheless Todt et al. showed that if the receiver is positioned at 90° and 9 cm from the external auditory canal or at 160°/9 cm, good visual assessment of the audiovestibular structures is feasible [6].

Retrospective analyses of implantees undergoing 1.5T scans showed a relationship between the specific MRI sequence and the assessment of the ipsilateral internal auditory canal [7]. Cochlear implant manufacturers have used different approaches to enable scanning procedures at 3T. Cochlear (Sydney, Australia), with a 3T-approved device [8], offers the option to remove the magnet as a solution for decreasing MRI-related artifacts. A similar solution is offered by Advanced Bionics (Stäfa, Switzerland), with approval for MRI scanning at up to 1.5T. Med-El (Innsbruck, Austria) recently introduced a device with approval for use at 3T using a magnet that makes removal unnecessary. Neurelec's device (Oticon Medical, Askim, Sweden) is approved for use at up to 1.5T, even with a removable magnet, but no solution has been offered with respect to the artifacts.

The aim of the present study was to observe differences in the magnet artifacts in relation to magnet position and MRI sequences under the visual assessment of the internal auditory canal and the labyrinth at 1.5T.

## 2. Material and Methods

All subjects have given their informed consent and the study protocol has been approved by the institute's committee on human research. The study was conducted according to the principles expressed in the Declaration of Helsinki. The receiver/stimulator (r/s) (Cochlear Nucleus freedom® dummy with magnet in place) unit was placed and fixed onto the head of three volunteers. The r/s was tightly fixed with a bandage to the head to avoid any displacement in nine different positions defined by the nasion–outer ear canal angle. The angles used were 90°, 120°, and 160° with a distance of the magnet from the outer ear canal of 5 cm, 7 cm, and 9 cm [6].

The volunteers were scanned with T1- and T2-weighted sequences at each of these nine positions. All examinations were performed in a 1.5 Tesla MR imaging unit (Ingenia, Philips Medical Systems, Best, NL) using an 8-channel array head coil.

An experienced neuroradiologist and two experienced neurotologists evaluated the internal auditory canal and labyrinth. The visibility of the internal auditory canal and labyrinth despite the artifacts produced by the magnet was graded as “not visible” (-), “good visibility” (+), and “excellent visibility” (++).

*Detailed MRI Scanning Parameters*. The parameters are as follows: TSE T1 2D: TR: 451* *ms, TE 9* *ms, slice thickness 1.5 mm, reconstruction resolution of 0.55 × 0.55 × 1.5 mm, F0V 130* *×* *140. 24 slices, and scan time of 3:22 minutes; and TSE T2 2* *D: TR: 3300* *ms, TE 120* *ms, slice thickness 1.5 mm, reconstruction resolution of 0.55 × 0.55 × 1.5 mm, F0V 120* *×* *120. 12 slices, and scan time of 2:50 minutes.

## 3. Results

**Figure 1:**
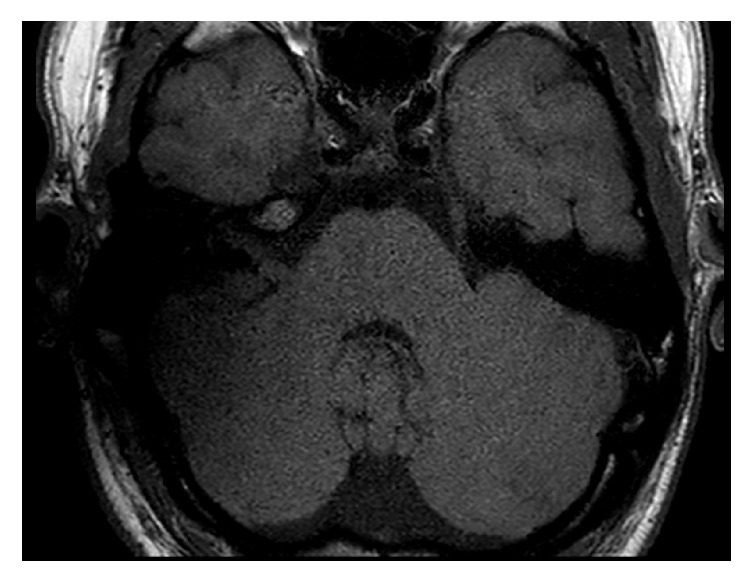
T1 sequence with the receiver/stimulator in a position of 90° and 9 cm. The IAC is good and the labyrinth is not visible.

**Figure 2:**
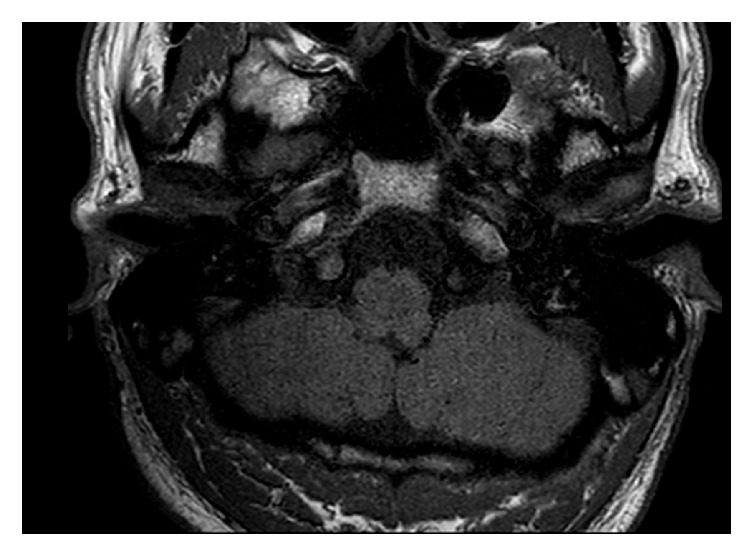
T1 sequence with the receiver/stimulator in a position of 120° and 9 cm. The IAC and the labyrinth are good visible.

**Figure 3:**
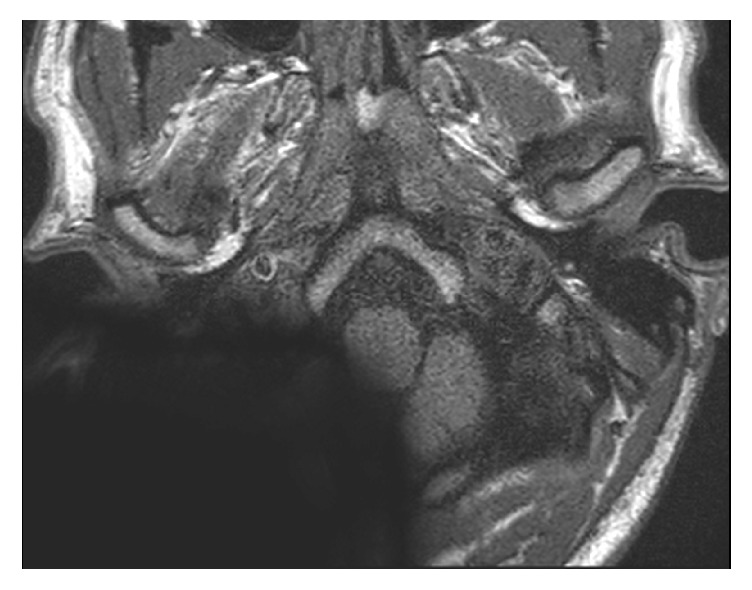
T1 sequence with the receiver/stimulator in a position of 160° and 9 cm. The IAC and the labyrinth are not visible.

**Figure 4:**
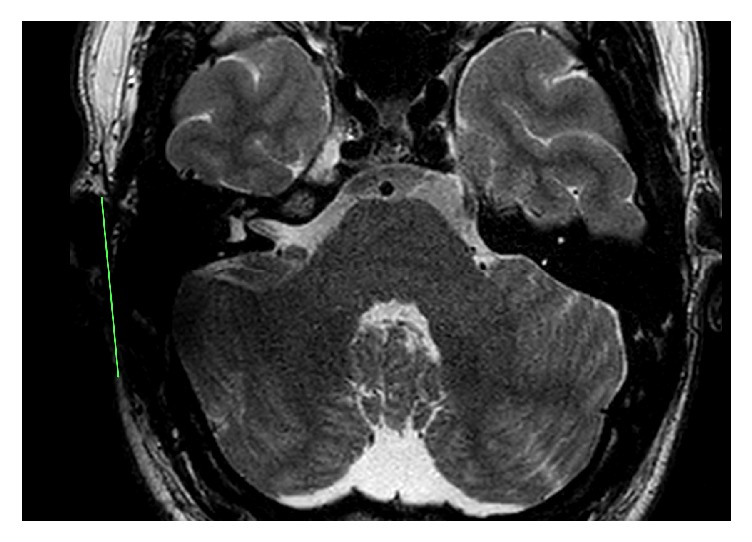
T2 sequence with the receiver/stimulator in a position of 90° and 9 cm. The IAC and the labyrinth are excellent visible.

**Figure 5:**
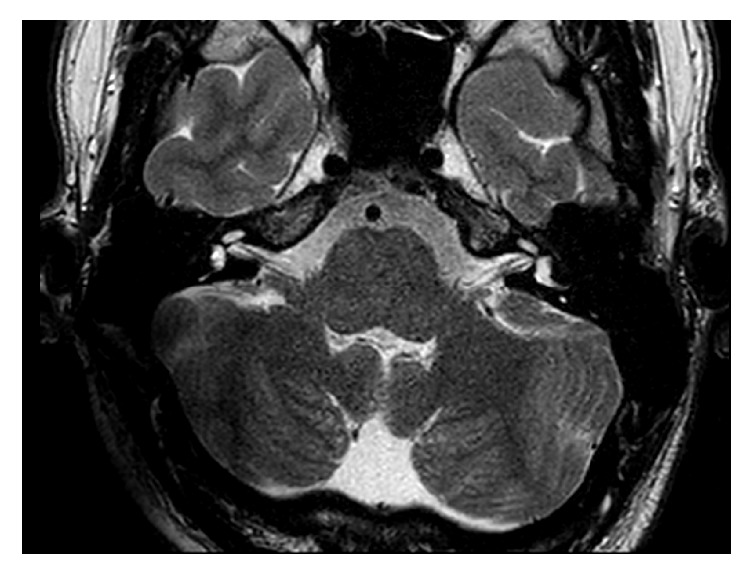
T2 sequence with the receiver/stimulator in a position of 120° and 9 cm. The IAC and the labyrinth are excellent visible.

**Figure 6:**
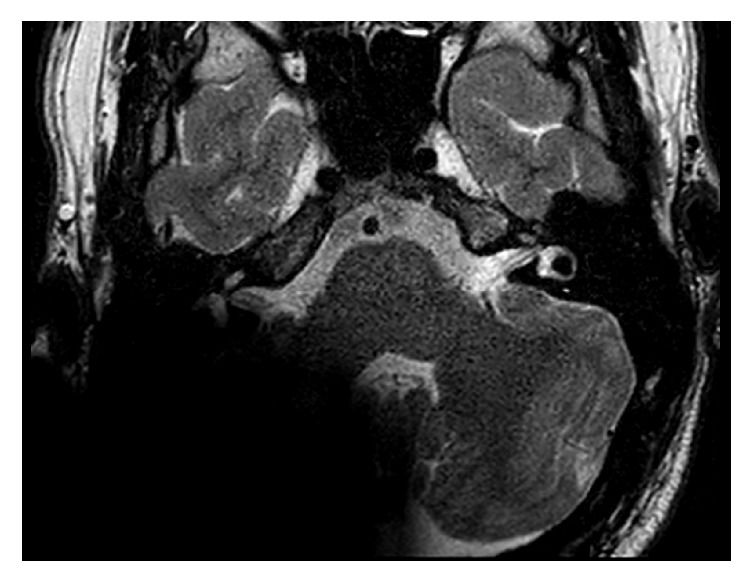
T2 sequence with the receiver/stimulator in a position of 160° and 9 cm. The IAC is excellent and the labyrinth is good visible.

The sizes of the skulls of the three subjects were between 56 and 57 cm and did not differ significantly from each other. In every subject the receiver/stimulator was placed on the right side. By comparing the different positions of the magnet, we could differentiate the labyrinth and the internal auditory canal (IAC) in relation to the magnet artifact when evaluating the scans. While the IAC and the labyrinth showed excellent visibility at 90° (T2, 9 cm distance), 120° (T2, 9 cm distance), and 160° (T2, 9 cm distance), the visibility was poor at 5 cm at all angles in T1 and T2 (Figures 1–6). At T1 the IAC and the labyrinth showed excellent visibility at 9 cm and 90° and 120° but were not visible at 160°. The labyrinth was good visible at 90° (7 cm and 9 cm distance). The subjects reported pressure on the side of the magnet but pain and displacement were denied.

## 4. Discussion

Cochlear implantation has become a standard procedure to rehabilitate patients with hearing loss worldwide. The indications are increasing and hence the number of recipients rises every year. Similar to cochlear implantation the number of MRIs is also rising in modern clinical practice.

MRI scanning with any hearing implant in place is a highly relevant issue. Scanning at 1.5T or 3T with cochlear implants in place causes artifacts that make large areas difficult to assess. To reduce the artifact produced by the receiver/stimulator, removable magnets are one way to overcome these issues. However, this approach bears the risk of wound and implant infection or subsequently even loss of the implant. Furthermore patients are required to undergo general anaesthesia for the removal and repositioning of the magnet. In the study by Wagner et al., cadaveric heads with implanted CIs underwent MRI scans at 1.5T and 3T with and without the magnet in place [9]. The artifact caused by the magnet is similar to our results in the 1.5T. Nevertheless they conclude that if the magnet is removed, MRIs at 1.5T and 3T can be done without sacrificing diagnostic imaging quality [9].

With evolving indications for cochlear implantation nowadays patients after vestibular schwannoma removal are enclosed. Postoperative visualization of the IAC and the labyrinth by MRI is of great importance for follow-up in these patients [10].

MRI scanning with the magnet in place may cause problems. Demagnetization is a potential problem, especially in 3T MRIs, and depends on the position of the magnet in relation to the magnetic field of the scanner. Implant displacement has not been widely discussed in the literature [11] as most relevant studies have focused on the visualization of the IAC and the inner ear [5].

In our previous study in a 3T MRI positioning of the magnet at 90°/9 cm and at 160°/ 9 cm allowed good visualization of the IAC and labyrinth structures. In MRI at 1.5T positioning of the magnet at 90°/9 cm, 120°/9 cm, and 160°/9 cm allowed good visibility of the IAC and labyrinth. In MRI at 1.5T the distance to the external ear canal is more influential than the nasion–outer ear canal angle. If the distance to the outer ear canal is reduced to 7 cm, excellent visibility is only possible at 120° (T2). Regarding the differences between sequences, better visibility with reduced artifacts can be achieved when using T2 weighted non-3D sequences. These findings are in line with Walton et al., who retrospectively analyzed 1.5T scanning sequences in NFII patients and also reported the possible visualization of residual tumor tissue in T2 weighted sequences.

External positioning of the magnet on the surface of the skin with a scalp thickness of about 6 mm had no significant impact on the visibility of the important structures in comparison to an implanted magnet. Our three volunteers had skull sizes of 56 and 57 cm and had similar and comparable outcomes. Significant differences might be seen in children as they have a smaller skull size.

Our study has some limitations. Only adults were included in our study. In children with smaller heads and hence a reduced magnet–IAC/labyrinth distance a bigger artifact with reduced visibility of the structures can be expected. Furthermore our results are based on the evaluation of only three adults. More patients would add more weight to our results.

In conclusion, with the visualization offered by MRI scans at 1.5T at specific magnet positions it is possible to exclude the recurrence of a vestibular schwannoma (neuroma) within the inner ear or the IAC if the distance and angle of the receiver/stimulator are appropriate. Furthermore it can be assumed that certain artifact-reduction algorithms will lead to greater tolerance limits with regard to angle and absolute distance of the implant.

## Data Availability

The MRI Images and DICOM files used to support the findings of this study are available from the corresponding author upon request.
